# Computational modeling of drug-eluting balloons in peripheral artery disease: Mechanisms, optimization, and translational insights

**DOI:** 10.1016/j.csbj.2025.08.005

**Published:** 2025-08-07

**Authors:** Mohammed A. AboArab, Vassiliki T. Potsika, Dimitrios S. Pleouras, Dimitrios I. Fotiadis

**Affiliations:** a Unit of Medical Technology and Intelligent Information Systems, Dept. of Materials Science and Engineering, University of Ioannina, Ioannina GR45110, Greece; b Electronics and Electrical Communication Engineering Dept., Faculty of Engineering, Tanta University, Tanta, Egypt; c Biomedical Research Institute, Foundation for Research and Technology-Hellas, University Campus of Ioannina, Ioannina GR45110, Greece

**Keywords:** Computational Modeling, Drug Delivery Optimization, Drug-Eluting Balloons (DEBs), Multiphysics Simulation, Patient-Specific Modeling, Peripheral Artery Disease (PAD)

## Abstract

Drug-eluting balloons (DEBs) represent a promising alternative to stent-based interventions for peripheral artery disease (PAD), and their therapeutic efficacy is dependent on optimizing drug transfer, mechanical deployment, and vessel—wall interactions. This review synthesizes current advancements in computational modeling; systematically analyzes studies identified through comprehensive ScienceDirect, Scopus, and PubMed (2015–2025) searches; and selects them according to PRISMA guidelines. Key strategies, including computational fluid dynamics (CFD), finite element analysis (FEA), fluid–structure interaction (FSI), and machine learning (ML), are critically examined to elucidate how drug kinetics, coating stability, and mechanical stress govern therapeutic outcomes. CFD-based mass transfer models capture flow-driven drug dispersion and washout dynamics, whereas FEA links balloon mechanics, plaque morphology, and drug penetration efficiency. FSI frameworks provide insight into the coupled effects of wall deformation and hemodynamics, identifying high-risk regions of drug underdelivery. ML-driven surrogates and physics-informed neural networks (PINNs) enable real-time, patient-specific predictions with computational accelerations exceeding 600 × while maintaining less than 2 % deviation from high-fidelity solvers. Persistent challenges include anatomical simplifications, limited *in-vivo* validation, and insufficient integration of biological remodeling. Future directions emphasize hybrid *in-silico* pipelines integrating imaging-derived 3D geometries, multiscale simulations, and AI-driven pharmacokinetic modeling to establish clinically translatable digital twins for precision-guided DEB therapies in PAD.

## Graphical Abstract

**Figure:**
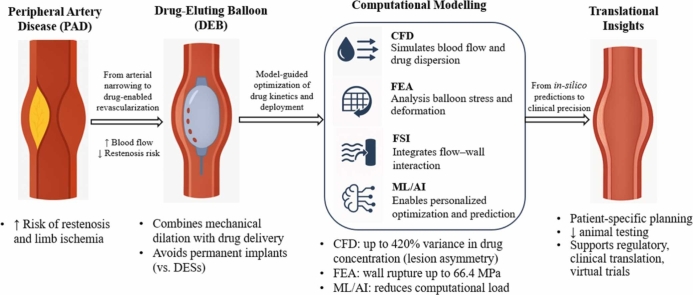

## 1 Introduction

Cardiovascular disease (CVD) is the leading cause of morbidity and mortality worldwide, affecting over 500 million people and accounting for approximately 20.5 million deaths in 2021 [1], [2]. Among the various forms of CVD, peripheral artery disease (PAD) is a prevalent and progressively increasing vascular condition characterized by atherosclerotic narrowing or obstruction of peripheral arteries, particularly in the lower limbs, resulting in reduced blood flow, ischemia-induced pain, fatigue, and impaired tissue perfusion [3]. A recent systematic review reported a greater than 17 % increase in PAD incidence over five years, equating to 30 million new cases and increasing the global burden to over 236 million individuals [4], [5]. PAD can manifest through intermittent claudication or remain asymptomatic, complicating early diagnosis. It significantly elevates the risk of cardiovascular events such as myocardial infarction and stroke, especially when combined with comorbidities such as diabetes or hypertension.

Conventional revascularization strategies such as surgical bypass, covered stents, and percutaneous transluminal angioplasty (PTA) have served as the cornerstones of PAD treatment [6]. However, these approaches frequently face challenges, including restenosis, stent thrombosis, and limited durability, especially in diffuse or complex lesions [7], [8]. Surgical bypass remains highly durable, with long-term patency exceeding 85 % in many cases [9], but it is invasive and associated with perioperative risk. Covered stents, while less invasive, have mixed long-term outcomes and may be prone to restenosis in small or calcified vessels.

Drug-eluting balloons (DEBs) and drug-coated balloons (DCBs) have emerged as promising alternatives to overcome these challenges. These devices combine transient mechanical dilation with localized delivery of antiproliferative agents, most commonly paclitaxel, to inhibit neointimal hyperplasia and enhance vessel patency. Unlike drug-eluting stents (DESs), DCBs avoid permanent polymeric scaffolds and their associated risks, such as chronic inflammation and delayed endothelial healing [10], [11], [12]. Clinical studies have shown that DCBs are superior to PTAs and non-inferior or even preferable to DESs in specific cases, including in-stent restenosis (ISR), bifurcation lesions, and small or calcified vessels [13], [14], [15]. Maximizing the therapeutic impact of DCBs requires a comprehensive understanding of balloon mechanics, drug release dynamics, vessel wall structure, and hemodynamic factors. While clinical and preclinical studies provide critical insights, computational modeling has emerged as a powerful complementary approach, enabling detailed simulations of drug transport, coating behavior, and device–artery interactions under patient-specific conditions [16], [17].

In particular, computational fluid dynamics (CFD) is used to model blood flow and drug diffusion; finite element analysis (FEA) is applied to study arterial wall stress, deformation, and balloon mechanics; and fluid—structure interaction (FSI) integrates both hemodynamic and structural responses [18], [19]. In addition, machine learning (ML) and artificial intelligence (AI) are increasingly used to optimize drug release parameters, identify influential design variables, and predict long-term outcomes on the basis of large datasets [20], [21]. Studies using advanced 3D simulations [22], [23] have explored drug transport behaviors during balloon deployment, accounting for key variables such as inflation time, arterial composition, and drug washout. Computational models have also been instrumental in predicting how coating microstructures influence drug transfer and retention, revealing critical factors that drive therapeutic efficacy. The convergence of CFD, FEA, FSI, ML/AI, and experimental or clinical data holds significant potential for advancing PAD treatments [23], [24], [25], [26]. These computational strategies deepen our understanding of drug delivery mechanisms and offer insights that inform the next generation of DEBs and DCBs with improved efficacy and safety profiles. Furthermore, these models pave the way for personalized treatment planning, enabling device customization and deployment strategies tailored to patient-specific vascular anatomy and pathology [27], [28], [29], [30], [31], [32].

Despite substantial progress, the application of computational modeling to DEBs remains less mature than that to DESs. Many studies still rely on simplified arterial geometries and uniform coating assumptions, which limit predictive accuracy. Recent advances in high-fidelity 3D modeling, hybrid *in-silico* and *in-vitro* approaches, and ML-driven predictive analytics have enhanced the reliability of simulations for long-term therapeutic outcomes. Coupling these methods with nanotechnology and experimental validation offers a powerful pathway toward the comprehensive optimization of drug-eluting devices, underscoring the need for further research to overcome existing challenges [33], [34], [35].

This review systematically evaluates the role of computational modeling in the design and optimization of DEBs for PAD treatment. We analyze the contributions of current modeling techniques, including CFD, FEA, FSI, and ML/AI-driven methods, to understanding device–tissue interactions and their potential to drive innovation in next-generation drug-eluting devices. Furthermore, we highlight ongoing challenges, emerging trends, and future research directions aimed at improving PAD outcomes through advanced computational strategies.

## 2 Methods

### 2.1 Review process

We conducted a systematic literature review to explore the breadth of computational modeling approaches applied to DEBs in the management of PAD. The review process included comprehensive searches in three major scientific databases: ScienceDirect [36], Scopus [37], and PubMed [38]. These platforms were selected for their extensive coverage of high-quality, peer-reviewed biomedical, engineering, and computational science publications. The search was restricted to publications written in English and published between 2015 and 2025 to reflect the evolving nature of computational tools and PAD intervention technologies. To ensure precision in data extraction and minimize bias, the inclusion criteria focused on original research studies involving computational techniques applied specifically to DEBs in PAD. The exclusion criteria included review articles, non-English publications, preprints, studies not related to PADs or DEBs, and those lacking sufficient methodological details. Each database was queried independently, and records were exported into a central repository for further analysis. Duplicate entries across the databases were identified and removed. A two-stage screening process was employed: initial title and abstract screening followed by full-text evaluation on the basis of predefined eligibility criteria. This process allowed for the categorization of relevant studies into distinct modeling approaches: CFD, FEA, FSI, and ML/AI-enhanced modeling.

### 2.2 PRISMA flowchart

**Fig. 1:**
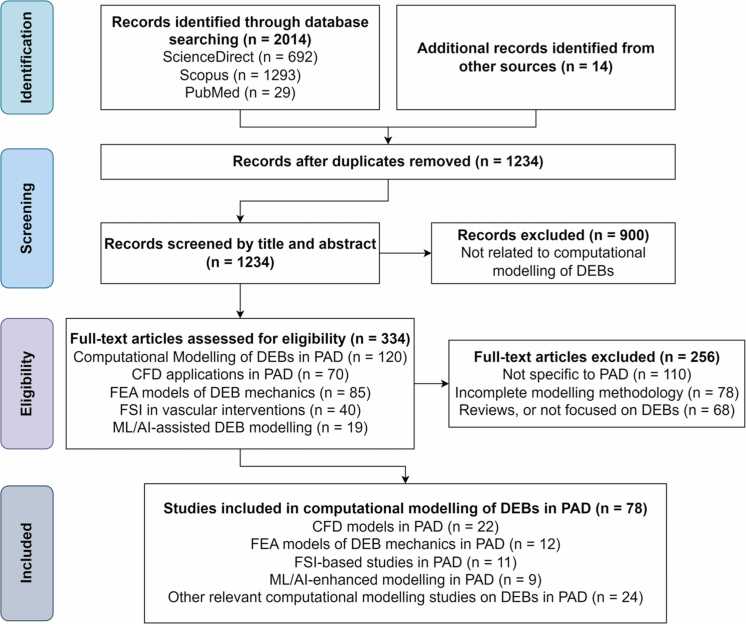
PRISMA flowchart illustrating the study selection process for the systematic review of computational modeling of drug-eluting balloons in peripheral artery disease.

The study selection process, illustrated in Fig. 1, followed the PRISMA guidelines [39]. A comprehensive search across three major databases, ScienceDirect (n = 692), Scopus (n = 1293), and PubMed (n = 29), identified a total of 2014 records, along with 14 additional records from other sources. After removing 780 duplicates, 1234 unique articles remained and were subjected to an initial screening on the basis of titles and abstracts. During this screening phase, 900 records were excluded because they did not meet the preliminary inclusion criteria, primarily because of a lack of relevance to computational modeling of DEB applications. In the eligibility phase, 334 full-text articles were assessed in detail against predefined criteria. Of these, 256 articles were excluded for one or more of the following reasons: not specific to PAD (n = 110), lacking computational modeling methodology (n = 78), being reviews, or not focused on DEBs (n = 68).

The remaining 78 studies that met all eligibility requirements were included in the qualitative synthesis. These studies were categorized into five primary computational modeling domains: CFD models in PAD (n = 22), FEA of DEB mechanics (n = 12), FSI studies (n = 11), ML and AI-enhanced modeling (n = 9), and other relevant computational modeling approaches involving DEBs in PAD (n = 24). This structured screening and eligibility assessment enabled the precise identification of computational methodologies applied to DEB-based interventions in PAD, forming a clear thematic foundation for the subsequent analysis.

### 2.3 Computational modeling pipeline for DEBs in PADs

**Fig. 2:**
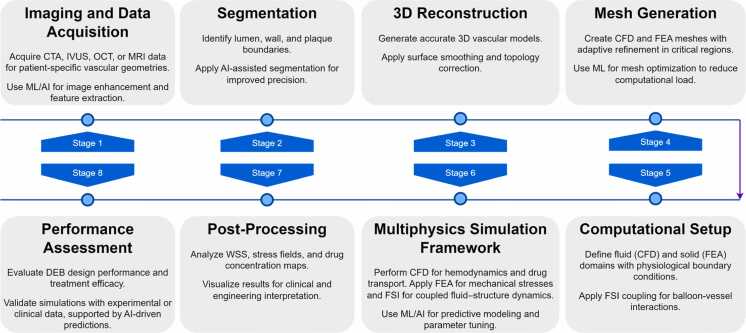
Computational modeling pipeline for drug-eluting balloons in peripheral artery diseases, illustrating key stages from imaging to performance assessment.

The computational modeling pipeline for DEBs in PAD consists of eight structured stages [40], [41], [42], [43], as illustrated in Fig. 2. (i) Imaging and data acquisition involve obtaining high-resolution computed tomography angiography (CTA), intravascular ultrasound (IVUS), Optical Coherence Tomography (OCT), or magnetic resonance imaging (MRI) datasets to capture patient-specific vascular geometries, with ML/AI techniques applied for image enhancement and feature extraction. (ii) Segmentation delineates the lumen, arterial wall, and plaque boundaries using semi-automated or AI-assisted methods to ensure anatomical accuracy. (iii) 3D reconstruction converts segmented datasets into detailed vascular models with surface smoothing and topology correction. (iv) Mesh generation produces high-quality CFD (fluid) and FEA (solid) meshes, incorporating adaptive refinement in critical regions and ML-based optimization for computational efficiency. (v) The computational setup defines the governing equations for CFD-based hemodynamics and FEA-based balloon—vessel mechanics, integrating fluid—structure interaction (FSI) for realistic deformation and flow coupling. (vi) The simulation framework combines CFD for flow and drug transport with FEA for structural analysis, which is enhanced by ML/AI models for predictive analytics and parameter tuning. (vii) Post-processing extracts and visualizes key outputs such as wall shear stress (WSS), stress–strain fields, and drug concentration maps for clinical and engineering interpretation. (viii) Performance assessment evaluates the mechanical behavior and drug delivery efficacy of DEBs, which has been validated against experimental or clinical datasets, with AI-driven sensitivity analysis to optimize treatment outcomes.

## 3 Applications of computational modeling of drug-eluting balloons

Computational methods have fundamentally enhanced DEB technology for PADs by offering precise insights into drug release, device—tissue interactions, and patient-specific responses [44]. Core techniques, such as CFD and FEA, model drug transport and structural dynamics to ensure optimal flow dynamics and mechanical stability, which are critical for drug efficacy under physiological conditions [45], [46]. Advanced fluid—structure interaction (FSI) models integrate CFD and FEA to simulate blood flow impacts on device stability, whereas patient-specific modeling and statistical optimization refine these models to reflect anatomical variability [47]. ML and AI contribute predictive power, enabling personalized treatment approaches by optimizing parameters such as dosage and coating properties [48], [49]. Together, these computational advancements are pivotal in developing DEBs with superior, patient-tailored efficacy, marking significant progress toward personalized PAD treatment.

### 3.1 Computational fluid dynamics

CFD is a pivotal tool for simulating blood flow and drug delivery dynamics under conditions such as PAD [47], [50]. Using finite volume analysis, CFD solves fluid dynamics equations, including the Navier—Stokes equations, and models drug transport through convection diffusion reaction (CDR) equations. The CFD process involves segmenting medical imaging data to create patient-specific *in-silico* arterial models, meshing the geometry, applying boundary conditions, and iteratively solving these equations to generate precise pressure and velocity profiles. This patient-specific approach allows CFD to simulate interventions such as balloon angioplasty or stent deployment, providing insights into drug interactions with vascular walls and facilitating dosage optimization.

In CFD analyses for incompressible flows, the Navier—Stokes and continuity equations govern mass and momentum conservation [51]:

(1) $$\partial \rho /\partial t\;+\;\nabla ∙\left(\rho *u\right)\;=\;0,$$

(2) $$\rho \left(\partial u/\partial t\;+\;u∙\nabla u\right)\;=\;-\nabla p\;+\mu \nabla^{2}u\;+\rho g,$$

where ρ is the fluid density, ∇ is the divergence operator, u is the velocity vector, p denotes the pressure, μ is the dynamic viscosity, and g represents the external forces [52]. Drug transport dynamics are modeled using the following CDR equation:

(3) $$\frac{\partial C}{\partial t}+\;\nabla ∙\left(u*C\right)\;=\;D\nabla^{2}C+R\left(C\right).$$

where C is the drug concentration, u represents convection, D is the diffusion coefficient, and R(C) accounts for biochemical reactions within the arterial wall.

The CFD modeling process includes acquiring anatomical data, performing digital segmentation, meshing, applying boundary conditions, running simulations, extracting data, and validating results against clinical benchmarks [53]. CFD has evolved from a tool for fundamental hemodynamic analysis to a critical enabler of DEB optimization, enabling precise evaluation of flow dynamics, drug transport, and device performance in patient-specific vascular environments [54], [55]. Early efforts focused on elucidating the role of WSS [56], the oscillatory shear index (OSI), and the relative residence time (RRT) as key determinants of plaque formation and vulnerability. Multiscale CFD frameworks, coupled with agent-based models (ABMs), provide insight into the interplay between localized hemodynamic forces and biological remodeling. These studies demonstrated that high atherogenic WSS zones could drive up to 80 % lumen reduction within two months, emphasizing the pivotal role of flow-induced mechanical forces in restenosis and disease progression [57], [58]. Building upon these foundations, patient-specific CFD models incorporating non-Newtonian blood rheology revealed that conventional Newtonian assumptions could underestimate low-shear regions by as much as 51 %, particularly in severely stenosed carotid and femoral arteries [59]. Non-Newtonian models, including Carreau-Yasuda and Quemada, improved the fidelity of WSS and OSI predictions, which remain robust indicators of plaque-prone regions. Parallel advancements in imaging-based CFD pipelines, which integrate CTA, IVUS, and 4D flow MRI, have significantly reduced the uncertainty in flow and pressure predictions, with segmentation and boundary condition refinements improving accuracy by up to 51 % in TAWSS estimation [58]. These hemodynamic simulations provide the groundwork for understanding the mechanical environment influencing drug delivery and uptake.

As the field has matured, CFD-based mass transfer models have become essential for quantifying drug distribution and retention in drug-eluting devices, particularly in stents and balloons. Detailed simulations of overlapping DESs revealed that drug concentration peaks in overlapping regions were 420 % higher than those in single-stent configurations, primarily because of flow recirculation and localized drug trapping [60]. This finding underscores the necessity of optimizing strut geometry and coating distribution to prevent cytotoxicity. Comparative analyses between durable polymer coatings and deployable crystalline coatings demonstrated that deployable coatings (e.g., MiStent) achieved 194–478-fold higher tissue drug concentrations and sustained 91 % retention for up to 90 days, compared with 21 % for durable coatings [61]. These insights guided the shift toward DEBs, which rely on short-term drug transfer during balloon inflation but require precise modeling of drug—tissue interactions for clinical efficacy. CFD-driven models of DCB performance confirmed that less than 5 % of the initial drug load penetrates the arterial wall during a 60-second inflation, with intimal diffusivity (1.67 × 10⁻¹¹ m²/s) and media binding kinetics (β = 10⁻⁴ s⁻¹) emerging as the dominant factors governing uptake [62]. These models further revealed that plaque heterogeneity, particularly the presence of necrotic cores or calcified regions, could reduce drug penetration by up to 100-fold due to reduced diffusivity and convective transport [63]. The Cleverballoon project integrated *in-vitro*, *in-vivo*, and CFD-based simulations to optimize everolimus-coated DCBs, combining OCT-derived 3D arterial reconstructions with coating stability and flow-mediated drug delivery modeling, thereby reducing the reliance on animal studies [64].

Validation and clinical translation have become cornerstones of CFD applications in DEBs [56], [65]. Hybrid 0D–3D Windkessel models, calibrated with 4D flow MRI, reduce flow rate prediction errors by 74–98 % and systolic pressure deviations by more than 20 mmHg, enabling highly accurate identification of hemodynamic hotspots in type B aortic dissections [66]. Clinical studies further leveraged CFD-derived pressure—flow indices (CPFD-caIMR), showing that DEBs are non-inferior to DESs for STEMI interventions while achieving lower major adverse cardiovascular event (MACE) rates over one year (OR = 2.98) [67]. These results highlight the transition of CFD from purely diagnostic modeling to direct clinical impact, where it informs device selection and procedural planning. Next-generation CFD methods integrate ML and physics-informed neural networks (PINNs) to accelerate high-fidelity simulations. These AI surrogates replicate velocity and pressure fields with less than 2 % error compared with Navier–Stokes solvers while offering computational speedups of over 600 × , making real-time prediction feasible [68]. Such advances are critical for simulating complex phenomena such as tracking-induced drug loss, where CFD and *in-vitro* studies have shown that up to 45 % of paclitaxel can be lost during device navigation, with 30 % loss occurring from a single arterial bend and significant off-target deposition in upstream vessels [40], [69].

Collectively, CFD studies (Table 1) highlight the dominant role of the WSS, OSI, and RRT in driving drug washout, plaque progression, and the performance of DEBs. Patient-specific geometries and non-Newtonian rheology models significantly increase prediction accuracy, particularly in low-WSS regions that are correlated with restenosis risk. However, key gaps remain, including the limited incorporation of FSI, the frequent use of rigid wall assumptions, and the lack of systematic calibration with longitudinal clinical data. While several studies have successfully combined CFD with mass transport models for drug elution [56], [60], [62], [63], there is a growing trend toward integrating hybrid 0D–3D models and AI-based surrogates [58], [62], [67] to achieve faster, clinically interpretable predictions. Overall, the CFD literature converges on the need for standardized imaging-to-simulation workflows, improved wall compliance modeling, and multiscale drug transport analysis to translate these findings into robust patient-specific treatment planning.

**Table 1:** Comparative overview of CFD-based studies on hemodynamics and drug-eluting balloons.

**Ref.**	**Computational Method**	**Quantitative Results**	**Clinical Relevance**	**Validation**
[57]	3D CFD + 2D ABMs	Lumen reduction up to 80 % in 2 months; sensitivity to α2 parameter	Predicts restenosis risk; evaluates angioplasty/stenting effectiveness	Qualitative histology comparison; multiple stochastic ABM runs
[59]	CFD with Newtonian vs Non-Newtonian rheology	Mean TAWSS difference < 6 % (Carreau/Cross) and up to 51 % for Quemada in low WSS zones	Identifies hemodynamic risk factors for plaque formation	Velocity profile comparison with PC-MRI; 15–61 % discrepancy noted
[68]	AI-enhanced CFD models (ML, PINNs)	ML models < 2 % error, 600 × faster computation; AUC 0.92 for CVD prediction	Real-time CVD risk prediction, plaque detection	Validated against CFD outputs and clinical datasets
[54]	Patient-specific CFD of aortic arch	High OSI at sinotubular junction; vortical and helical flows observed	Improves cerebral perfusion planning via rSCA cannulation	Limited validation vs 4D-MRI; qualitative assessment
[58]	Imaging-based CFD/FSI review	Segmentation errors cause 28–51 % TAWSS variation; compliant walls reduce TAWSS by 21.5 %	Low WSS as predictor for plaque initiation and vulnerability	Validated with 4D flow MRI (<4 % pressure waveform error)
[55]	CFD + FEA for device TPLC	Drag force analysis, clot capture efficiency, fatigue safety factors (FSFs) improved	Optimizes stents, IVC filters; reduces bench testing	ASME V&V40 framework for uncertainty quantification
[56]	CFD vs 4D Flow MRI	RNG k–ε model Pearson correlation up to 0.914 for velocities	Captures helical flows linked to pathologies	Voxel-wise MRI comparisons; p < 10⁻⁸
[56]	FSI on multi-plaque femoral artery	TAWSS 82.3 Pa (lipid) vs 20.8 Pa (calcified); VMS 118.7 vs 65.3 kPa	Assesses plaque rupture risk based on mechanical stress	Qualitative validation with literature trends
[65]	CFD + 4D flow MRI review	Optimized Fontan conduits reduce energy losses > 20 %	Non-invasive FFR computation improves diagnostic accuracy (>80 %)	Comparisons with PC-MRI and VFM
[60]	CFD + mass transfer modeling	420 % higher drug concentration in overlap zones	Highlights thrombosis and cytotoxicity risks	Grid independence and prior 2D study comparisons
[61]	DES drug release modeling	MiStent retains 91 % drug at 28–90 days vs 21 % for Xience	Supports deployable coating for reduced toxicity	LC-MS/MS quantification and IF imaging
[62]	Analytical drug transport in DCBs	< 5 % drug penetrates arterial wall; D₁= 1.67 × 10⁻¹ ¹ m²/s	Guides coating optimization for short inflation time	Convergence analysis (1000 eigenvalues)
[66]	0D−3D Windkessel + CFD	Flow error reduced by 74–98 %; systolic pressure drop by 22 mmHg	Identifies high-risk hemodynamic regions in dissections	Validated vs *in-vivo* 4D Flow MRI
[67]	CPFD-caIMR via CFD	DCB vs DES: MACEs OR= 2.98 at 1 year; CPFD-caIMR cutoff > 40 U	Non-invasive microvascular resistance prediction	Validated with clinical follow-up data
[64]	*In-vitro/in-vivo/in-silico* DCB modeling	OCT-based 3D reconstructions; preclinical healing studies	Optimizes everolimus-based DCB design	Animal models + computational validation
[63]	Patient-specific drug transport (DCB)	Drug retention varies ±47 % (soft vs hard models); 100 × lower diffusivity in NC/DC	Highlights plaque heterogeneity effects on drug uptake	Validated vs analytical & *in-vitro* zotarolimus data
[40]	Millifluidic setup + CFD	Drug loss ∼80 % at 1.7 Pa WSS; complete removal at 6.7 Pa within 30 s	Preclinical screening of coating stability	CFD verified WSS ranges
[69]	*In-vivo* + *in-silico* PCB tracking loss	44.5 % drug loss during tracking; 30 % at single arterial bend	Guides safer device navigation and coating improvements	Mass balance and LC-MS/MS validation

### 3.2 Structural analysis

Structural analysis, grounded in the FEA method, plays a critical role in understanding the mechanical behavior of drug delivery systems (DDSs), particularly DCBs and stents. FEA discretizes both the device and the surrounding tissue into smaller elements, enabling an elementwise analysis that predicts the overall structural response under applied mechanical forces, including stresses and strains. This method is essential for optimizing device design, ensuring mechanical durability, and assessing how interactions with the arterial wall influence drug release dynamics. The FEA process comprises meshing, application of boundary conditions, and assignment of material properties to each element, allowing researchers to iteratively solve governing equations for stress, strain, and deformation, ensuring both structural integrity and efficient drug delivery [44], [70].

The elasticity equation (stress—strain relationship) is described by the following equation:

(4) σ=Ε∙ε

where σ is the stress, Ε is Young’s modulus, and ε is the strain. The force—displacement relationship is formulated through a matrix equation [71]:

(5) K∙u=F,

where K represents the stiffness matrix, u is the displacement vector, and F is the force vector. This equation is central to determining the structural response of drug delivery devices under physiological conditions.

FEA has progressively advanced the understanding of DEB mechanics and drug transfer in PAD. Initial studies modeled balloon–artery interactions via semicompliant and noncompliant balloons with hyperelastic and bilinear elastoplastic laws, enabling the calculation of the lumen gain ratio (LGR) and elastic recoil ratio (ERR). The results revealed that increasing the inflation pressure from 10 to 14 atmospheres improved the LGR by approximately twenty percent (from 62 % to 83 % for lipidic plaques), with only a minor increase in the ERR (from 29.5 % to 30.5 %), demonstrating the importance of balloon sizing and pressure optimization [72]. Subsequent models introduced fracture mechanics to assess the predilation of calcified plaques. The simulations revealed that 270-degree calcified lesions fractured at pressures between 0.8 and 1.0 MPa, whereas 360-degree annular plaques resisted failure even at 1.4 MPa. Predilation improved stent roundness by approximately five percent (from 69.4 to 74.4 percent), highlighting how balloon type and lesion geometry determine procedural success [73]. Further developments in anisotropic hyperelastic damage models revealed that predilation to 3.2 millimeters increased final lumen expansion by 20–25 %, whereas noncompliant postdilation at 1.0 MPa achieved approximately eight percent additional lumen gain. These findings confirm that controlled rupture of plaques enhances lumen expansion but must be balanced against vessel injury risks [74].

The scope of FEA has expanded with drug transport modeling, where reaction–diffusion models simulate drug penetration across heterogeneous arterial tissues. For DEBs, the bound drug concentration remained at approximately 41 % of the initial dose after 25 h under zero-flux conditions, whereas it was only 10 % under zero-concentration conditions. Variations in fibrous cap diffusivity (from 6.23 × 10⁻⁶ to 6.23 × 10⁻¹⁰ square centimeters per second) reduced free drug peaks from 0.947 millimolar to 0.26 millimolar, revealing the dominant barrier effect of calcified plaques [75]. The incorporation of interstitial fluid flow and endocytosis further demonstrated that drug retention can reach 98 % under convective zero-flux conditions, with healthy tissue zones achieving nearly four times greater uptake than calcified zones [76]. Coating microstructure optimization has emerged as another focus area. A combined *ex-vivo*–FEA approach showed that increasing the content of hydrophilic excipients (such as urea or polyethylene glycol) at 15 % solid content improved paclitaxel transfer by ten-fold (urea) and five-fold (polyethylene glycol) compared with unmodified coatings. Adjusting the microstructural aggregation density (from one to eight aggregates per 100 micrometres) increased arterial wall drug uptake from approximately 40–60 % within 180 s [77]. Moreover, hybrid *in-silico–ex-vivo* studies linked balloon–artery contact pressure (CP) with drug transfer efficiency. CP varies from 0.16 to 0.35 atmospheres doubled drug transfer, increasing the mass from 30 to 60 micrograms, directly correlating mechanical deployment parameters with pharmacological outcomes [41]. Patient-specific FEA models further improved realism by incorporating plaque morphology and overstretching mechanics. Cohesive zone modeling predicted that critical stenosis greater than 15 % leads to early interlaminar failure at radii of 0.9–1.5 millimeters, whereas failure thresholds decrease from 286 % (for 7 % stenosis) to 119 % (for 64 % stenosis), underscoring the risk of over-expansion in peripheral vessels [78]. Digital workflows integrating OCT-derived geometries and ductile damage models have achieved lumen gain predictions with less than 15 % error, enabling virtual trials for peripheral angioplasty optimization [79].

Collectively, the reviewed FEA studies (Table 2) emphasize that the interplay between balloon mechanics, plaque morphology, and drug transfer is central to optimizing DEB performance [80], [81], [82]. The consistent correlation of the ERR and LGR with inflation pressures and lesion characteristics confirms their value as primary performance metrics [72], [73], [74]. These studies collectively show that calcification geometry and thickness are critical factors limiting lumen expansion and increasing the risk of vessel injury, whereas hybrid *in-silico–ex vivo* studies highlight the direct relationships among the CP, coating microstructure, and drug transfer efficiency [41], [77]. However, gaps remain, including limited modeling of asymmetric lesions, long-term vessel remodeling, and drug—tissue interactions under dynamic physiological conditions. Current trends point toward integrating patient-specific imaging data, multiscale material characterization, and coupled drug—mechanical simulations [75], [76], [78], [79], aiming to establish virtual testing frameworks for personalized DEB therapy planning.

**Table 2:** Comparative summary of FEA studies on drug-eluting balloons: computational methods, results, and clinical validation.

**Ref.**	**Computational Method**	**Quantitative Results**	**Clinical Relevance**	**Validation**
[72]	FEM with hyperelastic and elastoplastic plaque models	LGR increased ∼20 % (62 %→83 %) from 10→14 atm; ERR 28–36 %, peak strain 0.19→0.25	Optimizes balloon sizing and pressure for lumen gain	Validated against clinical ERR (32 % ±12 %)
[73]	FEA with brittle fracture modeling for calcified plaques	270° plaques fractured at 0.8–1.0 MPa; 360° plaques resisted fracture at 1.4 MPa	Guides balloon type selection for calcified lesions	Matches clinical thresholds for plaque fracture
[74]	FEA using anisotropic hyperelastic damage models	Pre-dilation (3.2 mm) improved lumen by 20–25 %; post-dilation at 1.0 MPa increased lumen by ∼8 %	Balances lumen gain with minimal vessel injury	Validated with *in-vivo* stress–stretch data
[75]	Reaction–diffusion modeling with VH-IVUS segmentation	Bound drug retention 41 % (zero-flux) vs 10 % (zero concentration) after 25 h	Reveals impact of plaque heterogeneity on DCB drug uptake	Calibrated with porcine and bench-top studies
[76]	Advection–reaction–diffusion with interstitial flow	Free drug retention 0.37 % (with convection) vs 0.22 % (without)	Highlights effects of convection and endocytosis on drug delivery	Validated with *in-vitro* drug washout data
[77]	*Ex-vivo* FEA of coating microstructure (UR/PEG excipients)	PTX transfer increased 10 × (UR) and 5 × (PEG) at 15 % excipient	Supports excipient optimization for DCB performance	Validated with SEM and LC-MS data
[82]	FEA of stent mechanics and drug diffusion	Von Mises stress 66.4 MPa (loaded); 3.7 % residual drug after 24 days	Links coating integrity to controlled drug kinetics	Validated with *in-vitro* drug release data
[78]	FEA with cohesive zone modeling for arterial rupture	Failure radius decreased 3.1→1.0 mm (7 %→64 % stenosis)	Identifies rupture risks in angioplasty	Consistent with clinical overexpansion data
[79]	Patient-specific FEA with ductile damage modeling	Lumen gain prediction < 15 % error	Enables patient-specific virtual trials	Validated with OCT lumen profiles
[81]	Mixed-dimensional FEA (1D stent, 3D artery/balloon)	Peak intima stress 757 kPa; 10 × faster simulations	Improves computational efficiency for virtual PCI	Validated with balloon expansion tests
[80]	FEA of IVL-induced cracks for stent expansion	Through defects increased lumen gain by 45–51 %	Optimizes IVL patterns for improved lumen area	Validated with OCT data
[41]	Hybrid FEM linking contact pressure with drug transfer	CP increase 0.16→0.35 atm doubled drug transfer (30→60 μg)	Optimizes balloon deployment parameters	HPLC and imaging validated coating transfer

### 3.3 Fluid-structure interaction (FSI) analysis

FSI analysis combines CFD and FEA to simulate the interactions between blood flow and the structural elements of drug delivery devices, such as stents or balloons, providing a holistic view of drug delivery dynamics and device stability under physiological conditions [83]. The process begins with CFD to calculate fluid parameters (velocity, pressure, and shear stress), which are then integrated with FEA to assess the structural response, enabling precise modeling of drug kinetics. FSI simulations require boundary and interface conditions to maintain the continuity of stress and velocity at the device—blood interface, with initial conditions capturing the physiological state of both fluid and structural elements [84].

The evolution of FSI modeling in vascular interventions has progressed from foundational hemodynamic studies to sophisticated, patient-specific frameworks that integrate drug kinetics, stent mechanics, and biological responses. Early efforts established baseline hemodynamic behavior under varying blood viscosities via 3D aortic models [85]. These simulations demonstrated that increasing blood viscosity from 0.005 to 0.1 Pa·s reduced velocity from 0.45 m/s to 0.18 m/s and lowered wall stress from 16,750 to 16,705 Pa, whereas aortic wall deformation decreased from 3.138 to 3.124 m. These findings emphasized the role of blood rheology in dampening flow and wall strain, offering early insights into conditions such as hyperlipidemia and thrombosis risk. The next stage of development involved patient-specific FSI modeling, where CT-derived geometries and physiological boundary conditions were used to optimize mechanical circulatory support devices such as VA-ECMO and IABP [86]. Coupled simulations revealed significant blood flow redistribution, with coronary perfusion increasing from 98.25 to 525.75 ML/min and carotid flow from 127.50 to 783.75 ML/min under combined ECMO–IABP support, whereas peak wall stress remained within 40–50 kPa. These studies marked a shift from generic modeling to real-world clinical planning, demonstrating the predictive value of FSI for device tuning and patient safety. A major advancement has been the integration of drug delivery modeling into FSI frameworks. Compared with conventional stents, bimodal diffusion models capture the interplay of drug release, flow patterns, and arterial wall motion, showing that optimized release profiles reduce diameter stenosis by 30 % and minimize the neointimal area [87]. In addition, DCB studies have incorporated plaque morphology and porosity, revealing that low-porosity calcified lesions decrease drug retention by 20 %, whereas extending balloon inflation from 15 to 30 s improves drug uptake by 15.5 % [88]. These results highlight the need for lesion-specific drug deployment strategies. The introduction of Navier–Stokes–Biot coupled models represented a rapid increase in accuracy, as these models integrated poroelastic wall mechanics and drug transport [89]. Simulations across multiple stent geometries (Onyx, Xience, Palmaz) revealed 30 % spatial drug concentration heterogeneity [90], [91] due to recirculation zones and local wall deformation—factors strongly linked to restenosis-prone regions. These models bridge the gap between mechanical performance and pharmacokinetics, guiding both stent design and drug release strategies.

FSI applications were further extended to stent biomechanics, where comparative analysis of *Cobalt-Chromium* (CoCr) designs highlighted the superiority of the Savior design, which exhibited the lowest WSS (51.4 Pa) and reduced radial stress, lowering the likelihood of endothelial damage [92]. Computational efficiency improvements, such as optimized boundary conditions (remote displacement vs. fixed support), reduce the runtime by 1.7–2 × without sacrificing accuracy, making FSI modeling more practical for design iteration. The latest generation of chemo-mechano-biological FSI models links hemodynamics, drug kinetics, and tissue healing. By coupling WSS patterns with neointimal hyperplasia, these models identified critically low WSS zones (0.01–0.08 Pa) near stent struts as hotspots for restenosis. Optimizing drug influx parameters suppressed hyperplasia but also delayed endothelial recovery, highlighting the trade-offs between drug efficacy and vascular healing [93].

Collectively, FSI studies (Table 3) illustrate that coupling fluid dynamics with wall mechanics and drug kinetics provides a more comprehensive and clinically relevant understanding of DEB and stent performance than fluid-only or structural-only approaches do. These models consistently highlight how dynamic wall deformation alters local hemodynamics, creating recirculation zones and low-WSS hotspots that strongly correlate with restenosis-prone regions [85], [87], [93]. While the inclusion of drug transport within FSI frameworks has advanced predictions of coating transfer efficiency and drug retention, the complexity of multiphysics coupling has limited widespread validation against long-term *in-vivo* data. Moreover, most current models simplify blood as having homogeneous Newtonian and arterial walls, potentially underestimating localized stress and diffusion heterogeneity. A clear trend is emerging toward patient-specific chemo-mechano-biological FSI simulations that integrate CT or OCT imaging, multistent geometries, and dynamic drug—tissue interactions [88], [93]. These advancements are enabling the transition from purely mechanical performance analysis to holistic, clinically actionable simulations capable of guiding lesion-specific drug deployment and stent design.

**Table 3:** Comparative summary of fluid–structure interaction studies in vascular interventions: computational advances, key findings, and clinical validation.

**Ref.**	**Computational Method**	**Quantitative Results**	**Clinical Relevance**	**Validation**
[85]	3D FSI model in COMSOL (laminar flow, 2.49 M elements)	Velocity 0.45→0.18 m/s; wall stress 16,750→16,705 Pa; deformation 3.138→3.124 m	Analyzes viscosity effect on perfusion and thrombosis risk	Mesh independence; comparison with published hemodynamic data
[86]	Patient-specific 3D FSI (ANSYS Fluent-Mechanical, CT-based)	Coronary flow 98.25→525.75 ML/min; carotid 127.50→783.75 ML/min; wall stress 40–50 kPa	Optimizes VA-ECMO/IABP for LV unloading and organ perfusion	Mesh independence (< 5 % error) and clinical data alignment
[87]	Bimodal diffusion + convection–diffusion–reaction modeling	Diameter stenosis reduced by ∼30 %; 88 % drug released in 1 day	Shows how drug release kinetics control restenosis	Validated with OCT and histological porcine data
[88]	2D axisymmetric FSI (FEniCS) with drug transport	Drug uptake 17 % at 10 min; 20 % reduction in calcified plaques; 15.5 % uptake gain (30 s vs 15 s inflation)	Guides DCB exposure time and plaque-specific drug transfer	Mesh independence (< 2 % error) and HPLC drug data
[89]	Navier–Stokes–Biot FSI with poroelastic walls and drug kinetics	30 % drug concentration variation near stent edges	Predicts drug underexposure zones; informs stent design	Compared with experimental drug release and wall deformation
[92]	Two-way FSI of 3 CoCr stents (anisotropic arterial walls)	WSS: SIMPLE 183 Pa, Bx_Velocity 85 Pa, Savior 51.4 Pa; 1.7–2 × faster using RD BC	Identifies optimal stent (Savior) with minimal restenosis risk	Cross-checked with WSS/OSI thresholds and literature data
[93]	Chemo-mechano-biological FSI (Navier–Stokes solver)	WSS baseline 0.8–1.5 Pa; critically low WSS 0.01–0.08 Pa near struts	Identifies ISR hotspots and drug-healing balance	Compared with coronary flow experiments and literature benchmarks

### 3.4 Machine learning and artificial intelligence

ML and AI have become integral in drug delivery research, enhancing drug release optimization and prediction accuracy for complex therapeutic outcomes [94]. By processing large datasets, ML and AI identify key parameters for precise drug targeting, enabling real-time adjustments and personalized treatment plans [95]. These methods simulate intricate drug release scenarios, optimizing variables such as coating thickness, release rates, and patient-specific factors, thus complementing traditional computational methods such as CFD and FEA. This fusion of data-driven models with conventional computational techniques yields a more flexible and accurate approach in drug delivery research. ML and AI are transforming DDSs by enabling sophisticated analysis of biological and chemical interactions, optimizing drug release, and supporting personalized treatment. AI-driven DDSs utilize sensor data for intelligent dosing adjustments, refining parameters such as dosage and timing to mitigate adverse effects. AI has also accelerated the 4D printing of responsive materials that adapt to environmental changes, offering innovative applications in DDSs. In addition, ML enhances drug discovery through virtual screening, expedites the identification of drug candidates and refines molecular targets via deep learning tools such as the automated hit identification and optimization tool (A-HIOT). Furthermore, ML predicts critical physicochemical properties, including solubility and permeability, reducing the number of experimental trials and guiding drug stability. These predictive capabilities extend to pharmacokinetics, enabling tailored DDSs that optimize efficacy while minimizing side effects, particularly in personalized medicine.

Recent advancements underscore the synergy between ML and computational modeling in advancing DCB technologies. Deep learning techniques, such as faster region-based convolutional neural networks (Faster R-CNN), have been applied to ultrasound imaging, demonstrating superior diagnostic accuracy for assessing DCB efficacy in arteriosclerotic occlusions, with strong agreement compared with digital subtraction angiography (DSA) [96]. Despite the limitations of small datasets, these approaches highlight the potential of ML for noninvasive, image-based performance evaluation. For device optimization, supervised ML algorithms have been proposed to reconstruct high-resolution simulations from coarse-mesh finite element data, reducing the computational cost while maintaining predictive fidelity [23]. While current implementations still lack full anatomical realism, this strategy markedly enhances the scalability of computational modeling pipelines. On the regulatory side, ML-driven data mining of real-world evidence and clinical trials has been leveraged for safety signal detection in paclitaxel-coated devices (PCDs), including both DCBs and DESs, enabling faster and more reliable decision-making [97]. From a materials design perspective, active learning and self-driving laboratory frameworks are being employed to accelerate the discovery of polymer-based drug delivery coatings. ML has been particularly effective in overcoming the curse of dimensionality by rapidly predicting performance metrics and guiding experimental workflows [98]. In addition, multiscale arterial models incorporating representative volume elements and FEA have been coupled with ML and AI algorithms to reduce computational burdens and achieve real-time, patient-specific simulations, which is critical for clinical translation [99]. Collectively, these studies demonstrate the growing role of ML across the DCB pipeline, from image analysis and mechanistic modeling to materials design and regulatory surveillance, while underscoring the need for robust validation and standardized data integration.

**Fig. 3:**
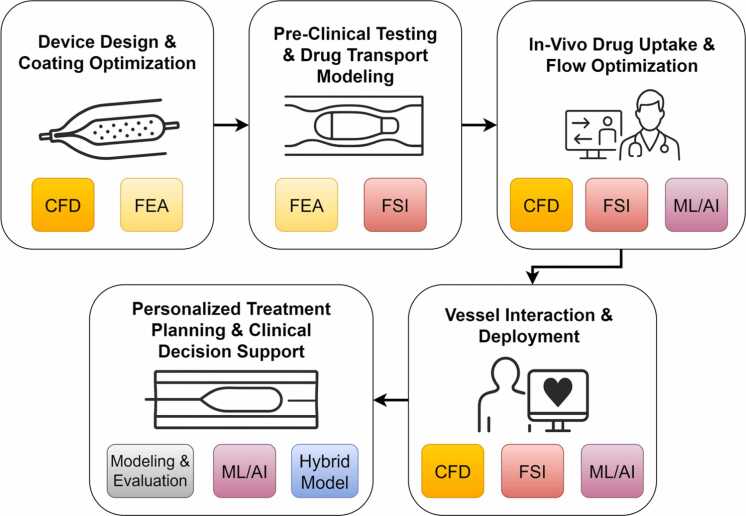
Holistic computational approach to drug-eluting balloon design and clinical support.

Advancing DEB technologies requires a comprehensive computational framework which combines all stages of the device lifecycle. Fig. 3 illustrates five key stages where computational modeling adds value, starting with (i) device design and coating optimization, where CFD quantifies drug coating uniformity and excipient transport under flow-induced shear stresses [57], [62], whereas FEA evaluates balloon material stress distributions and coating adhesion stability under varying inflation pressures [41], [74]. In the (ii) preclinical testing and drug transport modeling stage, FEA simulates drug diffusion across multi-layered arterial walls, and FSI captures coupled drug washout and wall deformation effects caused by transient balloon—vessel interactions [85], [93]. For (iii) *in-vivo* drug uptake and flow optimization, CFD and FSI provide hemodynamic metrics such as the WSS and OSI, whereas ML/AI-based surrogate models predict patient-specific drug retention and delivery efficiency [96], [98]. During (iv) vessel interaction and deployment, CFD models flow disruption and recirculation zones during inflation [62], FSI accounts for coupled fluid–structure dynamics, including recoil and plaque deformation [89], and ML/AI optimizes deployment strategies through real-time data-driven simulations [23]. Finally, in (v) personalized treatment planning and clinical decision support, hybrid modeling integrates CFD and FEA surrogates for rapid virtual prototyping [64], whereas ML/AI and modeling and evaluation frameworks enable risk stratification, outcome prediction, and personalized treatment planning [97], [100]. This integrated schematic underscores how the synergy between CFD, FEA, FSI, ML/AI, and hybrid modeling enables multiscale analysis, real-time predictions, and tailoring of DEB treatments to individual patient profiles.

## 4 Validation of computational models

### 4.1 Numerical validation

Numerical validation ensures the robustness of computational models through mesh independence, convergence studies, and time-step sensitivity analyses. For CFD models, grid independence checks (below 2 % variation in WSS predictions) and mesh refinement have confirmed the accuracy of velocity and shear stress predictions [54], [59]. FEA models use refined meshing and convergence testing on stress–strain distributions, reducing the number of numerical artifacts and ensuring the stability of the ERR and LGR outputs [72]. The FSI models combine both CFD and FEA numerical validation strategies, with high-resolution tetrahedral meshes (approximately 2.49 million elements) and time-step stability (0.005 s) confirming consistent velocity and pressure fields [85], [93].

### 4.2 Experimental validation

Experimental validation aligns simulation outputs with *in-vitro* and *ex-vivo* benchmarks, reinforcing the physical credibility of the models. CFD mass transport models have been validated using LC-MS/MS quantification and *in-vitro* drug release experiments, which successfully replicate the zotarolimus and sirolimus elution dynamics during balloon deployment [60], [63]. FEA models have been validated against mechanical balloon expansion and contact force measurements, where finite element-predicted pressures are strongly correlated with stamping force results and HPLC-based drug transfer assays ($R^{2}$ = 0.59, p = 0.0002) [41]. For FSI, bimodal diffusion models have been cross-validated with porcine drug retention data and OCT-based observations, confirming reductions of up to 20 % in drug uptake for calcified plaques [87], [88].

### 4.3 Statistical metrics

Quantitative validation metrics such as error margins, $R^{2}$ values, and p-values measure predictive fidelity. The CFD velocity profiles showed less than 15–20 % deviation from the PC-MRI velocity measurements, confirming the reliability of the flow and WSS distributions [56]. FEA models achieved ERR predictions of 32 % ± 12 %, aligning with angioplasty outcomes [72]. As described in the Experimental Validation section, hybrid FEA-drug transfer studies demonstrated strong correlation with HPLC drug measurements [41]. FSI models validated hemodynamic redistribution, where coronary perfusion improvements (from 98.25 to 525.75 ML/min) and carotid flow changes (from 127.50 to 783.75 ML/min) matched experimental and clinical trends [86].

### 4.4 Clinical validation

Clinical validation involves direct comparison with imaging and patient-specific datasets, ensuring the translatability of the models to real-world conditions. CFD velocity and WSS predictions have been benchmarked against 4D flow MRI and PC-MRI datasets [54], [59]. FEA models have been validated via VH-IVUS imaging and angiographic data, particularly for predicting plaque fracture thresholds and post-inflation lumen gain [75], [76]. FSI frameworks incorporating ECMO–IABP interactions have replicated clinical measurements of organ perfusion and LV unloading [86]. Drug-delivery models within both CFD and FSI have used patient-derived geometries to accurately evaluate drug transport and coating stability, ensuring clinical relevance [60], [63].

## 5 Discussion

### 5.1 The Role of computational modeling in DEB optimization

Computational modeling has emerged as a transformative enabler in DEB design [101], [102], [103], [104], evaluation, and personalization, bridging fundamental physics with clinical translation. As summarized in Table 4, these modeling strategies range from CFD-based hemodynamic analyses to FEM and FSI frameworks that incorporate realistic arterial geometries and boundary conditions. The evolution from simplified diffusion models to integrated multiphysics, patient-specific simulations has enabled predictive, evidence-backed insights into mechanical behavior, hemodynamics, and drug delivery kinetics. Among the most impactful approaches, CFD and 0D–3D coupled models, which are calibrated via advanced imaging methods such as 4D flow-MRI, have provided high-resolution mappings of WSS and perfusion distributions [66]. These non-invasive models minimize the need for catheter-based measurements while delivering physiologically accurate flow predictions, enabling precise simulation of transient drug transport during balloon inflation. For example, millifluidic CFD experiments have shown that up to 80 % of the therapeutic drug load can be lost during low-WSS tracking phases, with complete drug washout occurring within 30 s under 6.7 Pa WSS [40]. These insights have directly influenced excipient coating redesigns to improve drug retention during navigation.

**Table 4:** Computational strategies in biomechanical and structural modeling of drug-eluting balloons.

**Ref.**	**Computational Type**	**Geometry Type**	**Boundary Conditions**	**Validation Metrics and Their Values**	**Mesh Quality**	**Sensitivity Analysis**
[66]	CFD	Patient specific; MRI and CT reconstructed (healthy + dissected)	Inlet from 4D Flow-MRI; outlet calibrated 3-Element Windkessel (3EWM)	Cumulative least squares error reduced by 74–98 % vs. 4D Flow-MRI per branch	3.8 million elements; y + < 1; grid convergence ensured (TAWSS < 2.5 % variation)	Parameter perturbation ± 0.125–8 × to confirm convergence to physiologically relevant values
[105]	CFD	μCT-reconstructed porcine coronary artery with stent overlap	Flow rate inlet (0.95 ML/s), zero concentration at ostium, Murray’s law at outlets	Area-Weighted Average Concentration (AWAC): + 420 % (vs. proximal); Volume-Weighted Average Concentration (VWAC) confirms overlap peaks; 85 million element grid convergence	85 million tetrahedral elements; grid convergence verified	Segmental analysis (overlap vs. non-overlap); spatial variation of mural uptake
[61]	FEM	Idealized circular artery with embedded rectangular stent struts	Time-dependent boundary for dissolution; Dirichlet at strut-coating interface	> 98.5 % of deployed drug in crystalline form (predicted); matched LC-MS/MS drug load over 90 days	20k–36k Lagrange elements; COMSOL FEM with tight tolerances (10⁻⁶)	Evaluated dissolution rates based on crystal size and coating deployment extent
[67]	CFD	3D coronary artery reconstruction from angiography	Proximal pressure, distal pressure, diastolic flow velocity (Thrombolysis in Myocardial Infarction (TIMI) frame count); pressure drop from Navier–Stokes	Odds Ratio (OR): 2.95 (p = 0.03) for CV death/HF readmission with caIMR > 40 U; Receiver Operating Characteristic (ROC) Area Under the Curve (AUC) = 0.85	3D meshing via FlashAngio system; pressure integration over discrete grid	Multivariate regression of MACEs; ROC for time from symptom/door to balloon
[64]	FEM	Animal-specific 3D arterial reconstructions (OCT + angiography)	Inflation pressure + flow wash-out post-deflation; drug input from balloon contact zone	*In-vivo* OCT follow-up planned; pending quantitative validation data	Layered arterial wall with plaque heterogeneity; FEM meshing in 3D	Planned: DCB coating, excipient formulation, and dose simulations
[40]	CFD	Idealized femoral artery with 3D folded balloon model	Pulsatile inlet flow (0.577 m/s peak); zero-pressure outlet	WSS histograms match realistic systolic/diastolic ranges (1.7–13.4 Pa); High-Performance Liquid Chromatography (HPLC) quantifies 80–100 % drug loss under WSS	3D tetrahedral mesh (4–4.25 million elements); 8-layer boundary refinement	Evaluated WSS at multiple cardiac phases; flow viscosity variations for flow uniformity
[78]	FEA	Idealized artery with layered vessel wall and calcified plaque types	Displacement-driven balloon expansion; hard-contact; cohesive interlayer (traction-separation law)	Circumferential Vessel strain (CV) at failure: 109.8–298.4 %; critical stenosis for interlaminar rupture found between 15.4–36 %	C3D8R and C3D10M elements; S4R shell for balloon; validated material models with literature-based curves	Multiple plaque shapes (cylindrical/quarter); stenosis levels from 7–64 % tested for rupture response
[77]	FEM	Idealized 2D axial-radial coating–arterial wall cross-section	Initial concentration = 1 μM (coating); no-flux at base; open boundary elsewhere	↑ Aggregation → + 60 % PTX wall uptake in 180 s (vs. 30 %); LC-MS tissue drug concentration trends match	445,453 triangular elements; < 1 % error in drug mass transfer with refinement	Aggregate number (1–8 per 100 μm); matched *ex-vivo* trends in PTX transfer
[79]	FEA	OCT + angiography-based patient-specific bifurcated arteries	Fixed artery ends; pressure-driven balloon inflation (11–18 atm)	Lumen area error < 15 % vs. post-operative OCT; stent apposition detected for 5 cases	Hexahedral mesh via ANSA; layered structure; 2–4 elements per layer; Abaqus explicit solver	Validated 1 calibration case + 4 varied cases (stenosis 46–73 %, overlap, bifurcation)
[81]	FEM	Patient-specific coronary artery (OCT + Coronary Computed Tomography Angiography (CCTA)); layered wall structure	85 mmHg diastolic pressure; balloon inflation (13 atm); spring-supported ends	Stent recoil: 10.8 %; Lumen gain < 15 % error vs. post-operative OCT; Peak stress (757 kPa) near stenosis	714k+ DOFs (artery), beam-to-solid + mortar FEM; verified mesh effects; layered anisotropy	Curved vs. straight stenting; artery stiffness and stent types tested across geometries
[88]	FEM	Idealized and diseased arterial walls; patient-calibrated porosity	Flux input at balloon–wall interface; pressure gradient via Darcy’s law; post-angioplasty *J* = 0	10-min uptake = 17.02 % (vs. 14.4 ± 4.6 % *in-vivo*); mesh convergence < 2 %; 80 % free drug loss at 20 min	Gmsh + FEniCS; < 1 % variation with 20 % mesh density change	Porosity sweep (α); expansion time (15–60 s); binding/influx decoupling scenarios
[85]	FSI	Realistic aorta and branches (SolidWorks-based); time-varying inlet	Time-varying inlet pressure (initial: 126.09 mmHg); outlet sheds to arterial branches	Mesh convergence: best at 2.49 M elements; deformation stability at 3.12 μm; stress range: 16,705 Pa at μ= 0.1 Pa·s	Tetrahedral mesh; optimal with 2489,669 elements	Viscosity sweep (0.005–0.1 Pa·s); impact on velocity, pressure, deformation, stress
[92]	FSI	Idealized artery (SIMPLE, Boston Scientific Velocity Balloon Catheter (Bx_Velocity), Savior stents); layered adventitia-media-plaque	Fixed support vs. remote displacement (RD); Windkessel outlet; inlet velocity	WSS: 183 Pa (SIMPLE), 85 Pa (Bx_Velocity), 51.4 Pa (Savior); OSI/TAWSS/RRT validated spatially; RD vs. FS difference < 5 %	Hex20 (artery), Tet10 (plaque/stent), Prism6 (blood); 0.05–0.2 mm sizing; > 1.3 M elements	FS vs. RD BCs; short vs. long arteries; 3 stent designs (Simple, Bx_Velocity, Savior)
[93]	FEM	Patient-specific coronary artery (XIENCE-V) + idealized ring stent cases	Flow: pulsatile inflow via Fourier model; Wall: rigid (for now); Drug: time-varying strut flux	90-day simulation; drug effect delays reendothelialization; WSS2 validated over cycle; TAWSS: 0.8 Pa, OSI ≈ 0, RRT: 1.2–1.3 Pa⁻¹	Coronary model: 2.5 M tetrahedra; wall: hexahedral FEAP + bilinear elements; ALE-based WSS	Strut vs. ring design; Drug influx (qD); WSS1 vs. WSS2 error; SMC and collagen stretch variation

On the drug delivery side, CDR models have been instrumental in revealing how lesion-specific shear environments and tissue properties affect drug uptake. Patient-specific simulations demonstrated that fibrous tissues achieved 50 % receptor occupancy within one hour, whereas calcified regions exhibited only 7 % occupancy, acting as diffusion barriers [63]. Analytical CDR models have further indicated that less than 5 % of the initial drug payload penetrates the arterial wall within a 60-second inflation window, driving the development of coatings with enhanced transfer efficiency [62]. High-resolution 3D CFD combined with mass transfer modeling has also shown that overlapping stent struts alter recirculation zones, causing up to 420 % variation in local drug concentration, emphasizing the importance of uniform flow profiles during DEB deployment [60].

Finite element method (FEM) simulations complement these findings by linking mechanical deployment with drug kinetics. FEM frameworks have quantified lumen gain and vessel recoil, reporting ERRs between 28–36 %, which is consistent with clinical data, and LGRs of 51 % in calcified plaques vs. 62 % in lipidic plaques [72]. Increasing the balloon pressure (10–14 atm) improved the LGR by approximately 20 %; however, it also increased the degree of dissection risk due to increased peak arterial wall strain [72], [73]. Similarly, FEM-based microstructural modeling has demonstrated that optimizing the excipient aggregation density can double the acute drug transfer efficiency, whereas hybrid FEM-contact pressure studies have confirmed that balloon expansion pressures (0.16–0.35 atm) can double the coating transfer area and drug content (30–60 μg) [41], [77].

FSI modeling adds another layer of realism by coupling arterial wall mechanics with pulsatile blood flow. Advanced FSI studies have shown that patient-specific blood viscosity can significantly modulate wall stress and flow velocity (0.45 m/s down to 0.18 m/s as viscosity increases), which in turn impacts drug delivery performance [85]. Coupled *in-vitro–in-silico* studies have confirmed that low-porosity calcified plaques reduce sirolimus uptake by approximately 20 %, with optimal inflation durations of 30 s for maximal delivery efficiency [85], [88]. These findings have been clinically validated with porcine data, highlighting the translational relevance of such simulations.

In addition to physics-based modeling, ML and AI are increasingly being integrated into DEB pipelines to enable predictive analytics and personalized treatment planning. AI-enhanced CFD/FEA pipelines leverage imaging data (OCT, IVUS) to generate patient-specific models that predict lesion behavior, optimal inflation strategies, and drug uptake efficiency [23], [98]. For example, ML-driven surrogate models trained on simulation data have been used to rapidly estimate drug retention and mechanical stress patterns, significantly reducing computation times and supporting real-time clinical decision-making. AI-powered post-DEB imaging analysis has also shown potential for automated restenosis risk prediction and treatment optimization [96].

Hybrid pipelines, such as CleverBalloon, which integrates 3D FEM, CFD, and imaging-based reconstructions, exemplify the synergy between computational and experimental data. This approach not only optimized everolimus-coated balloon coatings but also reduced preclinical animal testing by providing validated virtual prototypes [64]. Moreover, multiscale models that couple arterial remodeling, drug kinetics, and flow perturbations [93], [99] demonstrate how *in-silico* studies can accelerate device iteration and regulatory evaluation.

### 5.2 Comparative insights from computational models: CFD, FEA, FSI, and ML

The combined body of work across CFD, FEA, FSI, and ML highlights that each modeling approach contributes a distinct but complementary perspective on DEB optimization. As summarized in Table 5, these computational models differ in their primary focus, strengths, and clinical relevance, with CFD excelling in hemodynamic analysis, FEA in mechanical deployment insights, FSI in holistic device–artery interactions, and ML in rapid, patient-specific predictions. CFD focuses primarily on hemodynamic forces such as the WSS, OSI, and RRT, which govern drug washout, plaque progression, and coating loss during device navigation [57], [58], [60], [62]. It quantifies flow-driven drug transport and reveals that factors such as intimal diffusivity (1.67 × 10⁻¹¹ m²/s) and media binding kinetics $\left(\beta =10^{-4}s^{-1}\right)$ dominate uptake during the 60-second inflation phase [62]. However, CFD alone neglects structural deformation and mechanical failure thresholds.

**Table 5:** Comparative analysis of computational models (CFD, FEA, FSI, and ML) for drug-eluting balloon optimization.

**Computational Model**	**Key Focus/Parameters**	**Strengths**	**Limitations**	**Clinical Relevance**
**CFD**	WSS, OSI, RRT, drug transport, flow patterns [57], [58], [60], [62]	Accurate hemodynamic and drug washout predictions [57], [60], [62]	Rigid wall assumption, no structural mechanics [54], [59]	Identifies drug washout zones and restenosis risks [60], [63]
**FEA**	ERR, LGR, contact pressure, plaque fracture [41], [72], [74]	Mechanical deployment insights, coating transfer [73], [74], [77]	No flow analysis, sensitive to material laws [75], [78]	Optimizes balloon sizing, inflation, and plaque treatment [41], [77]
**FSI**	Coupled WSS + stress, drug deposition, wall deformation [85], [88], [93]	Holistic device–artery interaction [86], [87], [89]	Computationally expensive, simplified blood/plaque models [85], [88]	Predicts both hemodynamics and drug retention [87], [88], [89]
**ML/AI**	Pattern recognition, surrogate modeling, predictive analysis [96], [97], [100]	Rapid prediction, real-time personalization [23], [96], [98]	Requires large datasets, less interpretable [97], [100]	Accelerates patient-specific simulation and coating optimization [96], [98], [100]

FEA, in contrast, captures the mechanical deployment of DEBs, where metrics such as LGR, ERR, and CP directly determine procedural success [41], [72], [74]. For example, increasing inflation pressure from 10 to 14 atm improves the LGR from 62 % to 83 % for lipidic plaques, whereas controlled plaque rupture during predilation increases lumen gain by up to 25 % but introduces vessel injury risks [73], [74]. These mechanical insights are essential for understanding the coating transfer efficiency and structural durability but lack the fluid-driven transport analysis presented in CFD.

FSI integrates both fluid and structural mechanics, providing a holistic framework for capturing how wall deformation influences local hemodynamics, drug deposition, and restenosis-prone regions [85], [93]. By modeling the coupling between arterial wall motion and blood flow, FSI reveals spatial drug heterogeneity of up to 30 % near stent edges due to recirculation zones [89] and highlights how lesion porosity and extended balloon inflation can improve drug uptake by more than 15 % [88]. These multiphysics models are closer to clinical reality but remain computationally demanding and rely on simplifying assumptions such as Newtonian blood flow or homogeneous plaque material.

ML and AI bridge these gaps by acting as surrogates for high-fidelity simulations and enhancing predictive capabilities. They enable rapid reconstruction of high-resolution FEA and CFD fields with less than 2 % error [23] and leverage imaging data (e.g., Faster R-CNN applied to ultrasound) to achieve high diagnostic accuracy of DCB performance compared with DSA [96]. In addition, ML-driven material discovery accelerates coating optimization and integrates multiscale datasets for real-time, patient-specific predictions [98], [100].

In terms of clinical relevance, CFD excels at predicting drug washout and flow-induced risks, FEA is critical for mechanical optimization, FSI provides the most physiologically realistic insights, and ML enhances scalability and personalization. These approaches are complementary rather than conflicting, with hybrid pipelines increasingly combining CFD/FEA simulations with ML-driven parameter optimization and FSI frameworks for comprehensive virtual trials.

### 5.3 Challenges, limitations, and future work

Computational modeling has revolutionized the optimization of DEB technologies; however, critical challenges and emerging trends continue to shape its translational potential. The primary difficulty lies in accurately replicating patient-specific hemodynamics, especially in PAD, where arterial morphologies, plaque heterogeneity, and flow patterns vary significantly among patients. CFD models calibrated with 4D flow-MRI offer high-resolution insights into WSS and perfusion but are constrained by rigid-wall assumptions, simplified Windkessel boundaries, and limited MRI resolution, which reduce physiological accuracy under pulsatile and tortuous flow conditions [54], [56], [59], [66]. Integrating FSI models with multi-VENC MRI and real-time one-dimensional solvers is an emerging trend that could dramatically enhance anatomical fidelity and dynamic modeling capabilities.

CDR models remain computationally efficient tools for early-phase device testing but rely on idealized radial symmetry and homogenous arterial layers, excluding essential biomechanical factors such as plaque stiffness, wall compliance, and inflation-induced stresses [62]. Recent trends emphasize hybrid approaches, which combine these analytical cores with AI-driven surrogate models and PINNs to accelerate sensitivity analyses and enable adaptive, patient-specific DEB optimization [58], [68]. This synergy of physics and ML is increasingly viewed as a transformative direction for personalized vascular therapies.

CFD–mass transfer studies have revealed that geometry-induced recirculation zones, particularly near overlapping stents, can drive local drug concentration variations exceeding 420 % [60]. However, most computational frameworks omit key biological mechanisms such as inflammation, endothelial regeneration, and vascular remodeling, limiting their predictive power for *in-vivo* drug kinetics. The trend is shifting toward multiscale pharmacodynamic models, which integrate cellular-level drug uptake and time-resolved tissue responses, augmented by ML-enhanced pharmacokinetic predictions [61]. FEA-based studies, which are critical for modeling balloon mechanics and drug depot dissolution (e.g., MiStent), are still limited by 2D geometries and oversimplified lesion modeling [61], [73], [74]. The future lies in combining high-fidelity 3D reconstructions from OCT/IVUS imaging with AI-driven structural analysis to build digital twins for pre-procedural planning [75], [76], [79].

A critical but often underexplored issue is drug loss during catheter navigation, which can exceed 30 % in tortuous vessels [106]. Existing models rarely capture catheter—vessel interactions, dynamic wall deformation, or coating fragmentation, all of which directly impact drug delivery efficiency. Future computational pipelines should integrate mechanical–chemical coupling and leverage ML models to predict real-time catheter tracking and coating degradation. Similarly, indices such as coronary angiography-derived microcirculatory resistance (caIMR) [67] require validation in PAD settings, as differences in vascular structure and lesion composition complicate direct translation.

Advanced frameworks such as CleverBalloon [64] demonstrate the potential of end-to-end DEB simulation, but they remain limited by animal-derived geometries, simplified transport assumptions, and the absence of cellular-scale modeling. For clinical translation, incorporating patient-specific plaque morphologies, post-inflation drug washout dynamics, and molecular-scale interactions is essential. Although high-fidelity FSI models [87], [92] offer deep insight into vascular deformation and flow interactions, their high computational cost restricts scalability. The trend toward AI-driven surrogate modeling, which can accelerate simulations by more than 600 × with less than 2 % error, is opening pathways for near-real-time digital twin-based decision support [58], [68].

Finally, multiscale and ML-based frameworks redefine the DEB research landscape by linking microstructural parameters (e.g., collagen orientation and smooth muscle density) to macro-scale hemodynamics and drug transport [99]. However, challenges such as the lack of standardized modeling protocols, limited multi-center datasets, and insufficient FAIR data principles [55], [58] hinder their clinical adoption. Future trends must prioritize hybrid, multiphysics pipelines that combine CFD, CDR, FEA, and FSI with AI-enhanced pharmacokinetics and regulatory validation, supported by biological signaling pathways (e.g., TGF-β and Notch signaling). The convergence of ML, advanced imaging, and physics-based models will drive computational modeling from a research tool to a regulatory-grade, clinically actionable system for personalized PAD therapies.

## 6 Conclusion

Computational modeling has become a key tool for optimizing drug-eluting balloons (DEBs) in peripheral artery disease (PAD). This study provides precise insights into drug transfer, balloon mechanics, and vessel interactions, complementing experimental and clinical studies. This review highlights how advanced modeling frameworks that include computational fluid dynamics (CFD), finite element analysis (FEA), and fluid–structure interaction (FSI) have unraveled the complex interplay between arterial geometry, mechanical stresses, and drug transfer kinetics. CFD studies have quantified flow-driven drug dispersion and identified recirculation zones where local concentrations can vary by more than 400 %, whereas FEA has revealed critical correlations between balloon mechanics, plaque morphology, and coating stability. Coupled FSI models offer physiologically realistic predictions of arterial wall deformation and drug–wall contact, directly guiding device design. Machine learning (ML) and physics-informed neural networks (PINNs) have further increased modeling efficiency, reducing the reliance on computationally expensive simulations while enabling accurate patient-specific predictions.

Key trends indicate a shift toward multiscale, hybrid modeling pipelines, where cellular-scale drug uptake and tissue remodeling are integrated with macroscopic hemodynamics and mechanical responses. Emerging digital twin approaches, powered by imaging-derived 3D geometries and AI-driven pharmacokinetic models, are poised to deliver real-time, patient-specific treatment optimization. However, challenges persist, including anatomical oversimplification, limited *in-vivo* validation, and the absence of standardized modeling protocols, which hinder clinical translation. Future work should focus on incorporating biological signaling pathways, mechanical–chemical coupling, and large-scale, multi-center datasets to develop clinically validated *in-silico* platforms. The convergence of high-fidelity physics-based simulations, AI-driven analytics, and experimental validation promises to advance DEBs into a new era of precision-guided interventions for PAD.

## CRediT authorship contribution statement

**Mohammed A. AboArab:** Writing – review & editing, Writing – original draft, Visualization, Validation, Methodology, Investigation, Conceptualization. **Vassiliki T. Potsika:** Writing – original draft, Methodology, Investigation. **Dimitrios S. Pleouras:** Writing – original draft, Methodology, Investigation. **Dimitrios I. Fotiadis:** Writing – review & editing, Writing – original draft, Supervision, Funding acquisition, Conceptualization.

## Declaration of Competing Interest

The authors declare that the research was conducted in the absence of any commercial or financial relationships that could be construed as potential conflicts of interest.
